# Account of Diseases in an Eastern District of London, from July 20 to August 20, 1804

**Published:** 1804-09-01

**Authors:** 


*Account of Diseases in an Eastern District of London, from July 20 to August 20, 1804.*

| | |
|---|---|
| ACUTE DISEASES. | |
| Pneumonia | 3 |
| Hepatitis | 2 |
| Rheumatismus Acutus | 7 |
| Cholera | 6 |
| CHRONIC DISEASES. | |
| Tussis | 12 |
| Dyspnœa | 7 |
| Tussis cum Dyspnœa | 14 |
| Phthisis Pulmonalis | 6 |
| Hydrothorax | 4 |
| Ascites | 3 |
| Scirrhus Uteri | 1 |
| Prolapsus Ani | 2 |
| Cephalalgia | 7 |
| Gastrodynia | 6 |
| Hysteria | 5 |
| Hypochondriasis | 4 |
| Chlorosis | 6 |
| Scrophula | 3 |
| Herpes | 4 |
| Rheumatismus Chronicus | 12 |
| PUERPERAL DISEASES. | |
| Peritonitis | 3 |
| Menorrhagia Lochialis | 5 |
| Ephemera | 5 |
| INFANTILE DISEASES. | |
| Aphthæ | 4 |
| Herpes | 3 |
| Ophthalmia | 3 |
| Diarrhœa | 4 |

The autumn now advancing, the diseases peculiar to that season begin to shew themselves.

Several instances of cholera have occurred. This disease, which most frequently makes its appearance in the autumnal months, has been attributed to effects produced on the hepatic system during the heat of the summer. These are more particularly observable when a copious fall of rain has succeeded a very warm temperature of the atmosphere.

As this disease is endemic in the warm countries, it is natural to ascribe it to an increased degree of heat, and it has accordingly been observed to prevail more or less in proportion to this.

The more immediate cause of this disease is to be found in the morbid action of the liver.

Dr. Saunders supposes, from the quantity of bile secreted, and the rapid manner in which it is poured into the duodenum, "that there is not time sufficient for a perfect secretion, but that the fluid is somewhat of an intermediate nature between blood and bile;" and that "a considerable quantity quantity of red globules escape, unchanged, from the capillary vessels into the pori biliarii, and, uniting with a portion of bile, are carried by the hepatic ducts into the duodenum."

In the treatment of this disease, emetics and cathartics seem unnecessary, unless, indeed, by too early or too free a use of opiates, the evacuation from the intestines has been prematurely checked, and great pain in the bowels ensues in consequence of this, when the use of the latter may be highly necessary.

Although too early an use of opiates may prove injurious, the well directed use of them is highly salutary, especially in those cases where spasms of the lower extremities prevail. It is highly proper throughout the disease to dilute the contents of the stomach and intestines by the use of some emollient fluid, and also in some cases to throw up a glyster.

